# Supplementary material for: Contribution of the medial eye field network to the voluntary deployment of visuospatial attention
Source: Nat Commun. 2022 Jan 17;13:328. doi: 10.1038/s41467-022-28030-3 (PMC8763913; doi:10.1038/s41467-022-28030-3)
Supplement: Supplementary file 1 — Supplementary Information [file 41467_2022_28030_MOESM1_ESM.pdf]

**SUPPLEMENTARY INFORMATION**

# Contribution of the medial eye field network to the voluntary deployment of visuospatial attention

Guillaume Herbet[1.2*]. Hugues Duffau[1.2]

## Summary

### 1- Supplementary Figures

**Supplementary Figure 1:** Density plots showing the data distribution of healthy participants *versus* patients at each assessment
**Supplementary Figure 2:** Proportion of individual deficits at each assessment for the three behavioral measures of interest
**Supplementary Figure 3:** Grid search for SVR-LSM models of *A1* and $\Delta 2$
**Supplementary Figure 4:** Grid search for SVR-LSM models of $\Delta 1$
**Supplementary Figure 5:** Results from group analyses for total_bell
**Supplementary Figure 6:** Native FLAIR MRI of the unique patient

### 2- Supplementary Tables

**Supplementary Table 1**: Sociodemographic and clinical data for the 128 patients
**Supplementary Table 2**: Correlations analyses between the behavioral measurements of visuo-spatial attention and the demographic/clinical data
**Supplementary Table 3**: Longitudinal behavioral data for the 128 patients
**Supplementary Table 4:** Demographic and behavioral data for the 44 healthy participants
**Supplementary Table 5**: Comparisons between controls and patients at each assessment
**Supplementary Table 6**: Between-task correlations
**Supplementary Table 7**: Proportion of parietal and MFC patients with a new deficit after surgery

### 3- Supplementary analyses

### 4- Supplementary Notes

Supplementary Note 1
Supplementary Note 2
Supplementary Note 3

### 5- Supplementary references

**1- Supplementary Figures**

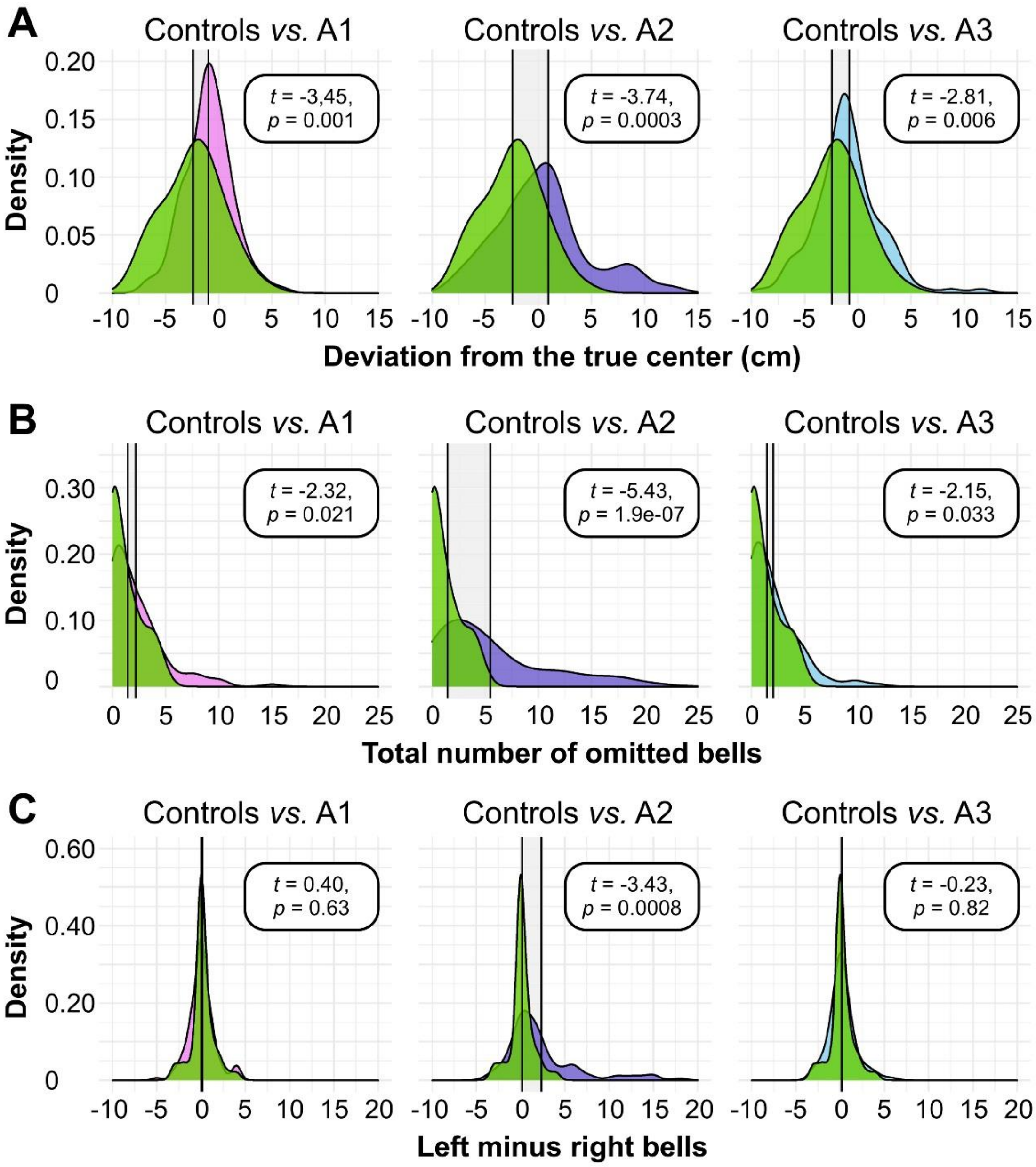


**Supplementary Figure 1:** Density plots showing the data distribution of healthy participants (in green) (n = 44) versus patients (n = 128) at each time point (A1, A2, A3) for line bisection estimates (**A**), total_bell (**B**) and diff_bell (**C**). Vertical lines represents the mean of each distribution. *t*-tests for independent samples were used to assess statistical significance. See Supplementary Table 5 for details about the statistical analyses A1, preoperative assessment; A2, 5-day postoperative assessment; A3, three-month postoperative assessment. R software (https://www.R-project.org/; packages = ggplot2 & ggpubr) was used to create this figure. Source data are provided as a Source Data file.

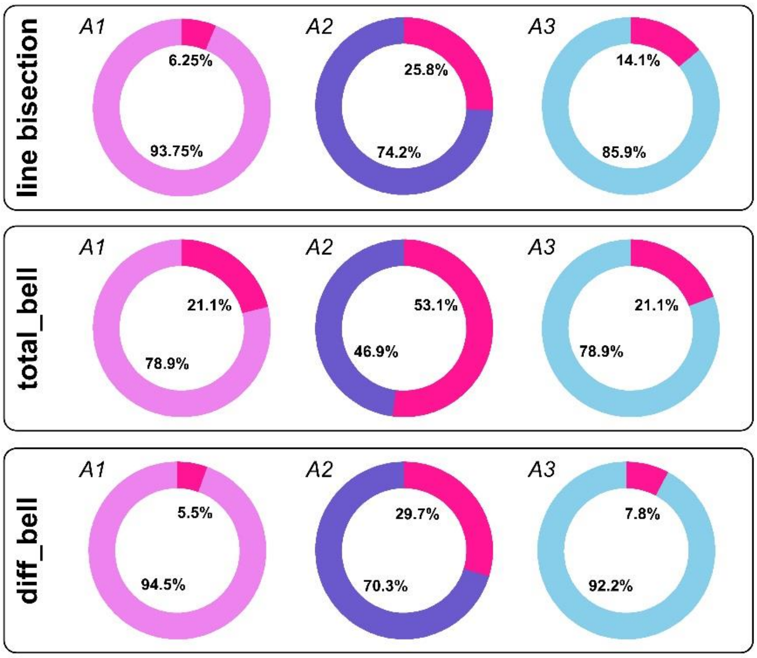


**Supplementary Figure 2:** Proportion of individual deficits at each assessment for the three behavioral measurements of interest. Z-scores were computed based on the normative distributions of healthy participants. A score was considered as impaired when the corresponding z-score was equal or superior to 1.65 (p = 0.05 one-tailed). As shown in this figure, the rate of deficits at A1 was very close to what it would be expected in the normal population (i.e. 5%), especially for line bisection estimates and diff_bell – the two direct measures of neglect in this study. R software (https://www.R-project.org/; packages = ggplot2 & ggpubr) was used to create this figure. Source data are provided as a Source Data file.

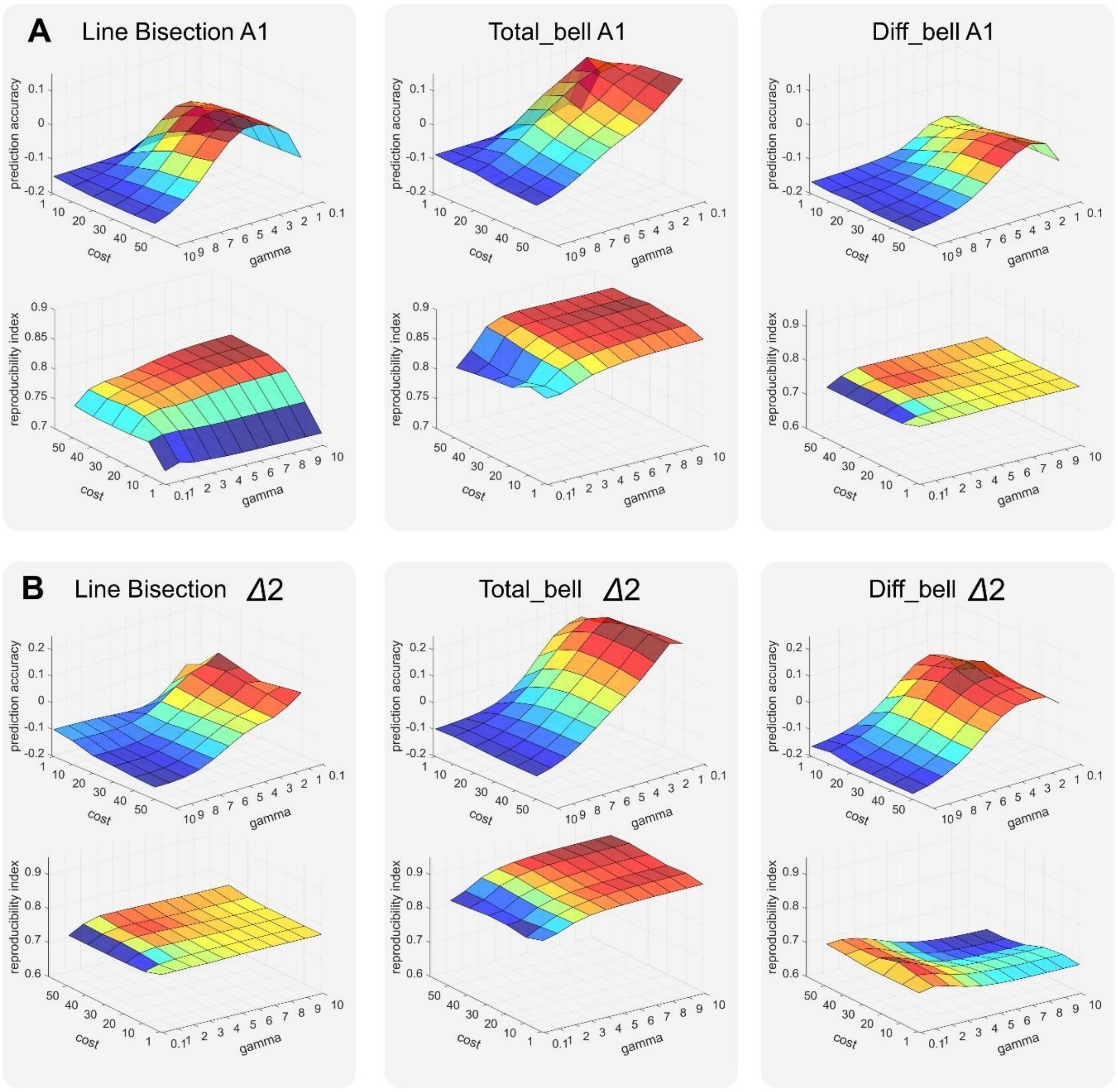


**Supplementary Figure 3:** Grid search for SVR-LSM models of A1 **(A)** and Δ2 **(B)**. As shown in this figure, prediction accuracy and/or reproducibility were not enough to generate reliable lesion-symptom maps. Note that the directionality of the x- and y-axis is reversed for prediction accuracy. The plots were created with Matlab's surface plot function. Source data are provided as a Source Data file.

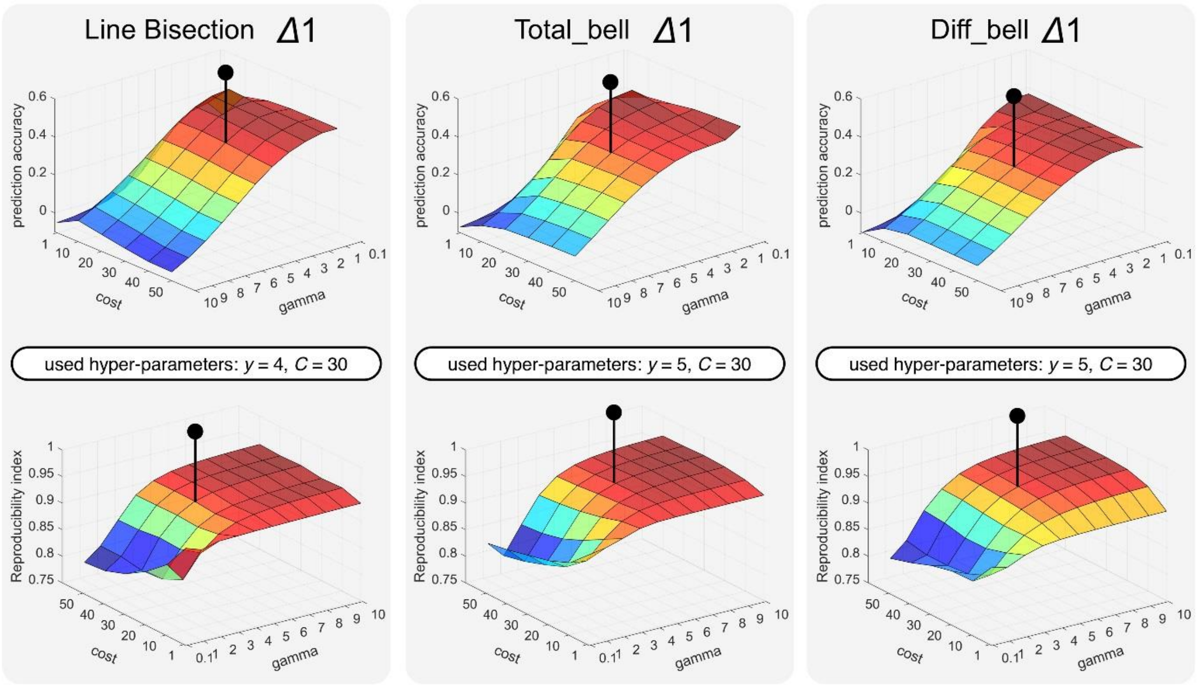


**Supplementary Figure 4:** Grid search for SVR-LSM models of Δ*1*. The retained hyper-parameters are indicated on the figure. Note that the directionality of the *x*- and *y*-axis is reversed for prediction accuracy. The plots were created with Matlab's surface plot function. Source data are provided as a Source Data file.

**Supplementary Figure 5:** Results from the group analysis for total_bell. A mixed ANOVA (two-sided) was conducted to assess statistical significance, with time of assessment {A1, A2, A3} as a within-subject factor and patient group as a between-group factor {parietal, MFC}. A principal effect was found for *assessment time* ($F_{(2, 74)}$ = 46.62, $p < 0.001$, $\eta^2_p = 0.56$; two-sided) but not for *group* ($F_{(1, 37)}$ = 0.0005, $p = 0.98$, $\eta^2_p = 0$; two-sided). Both factors did not interact significantly ($F_{(2, 74)}$ = 0.71, $p = 0.49$, $\eta^2_p = 0.018$; two-sided). R software (https://www.R-project.org/; packages = *ggplot2* & *ggpubr*) was used to create this figure. Source data are provided as a Source Data file.

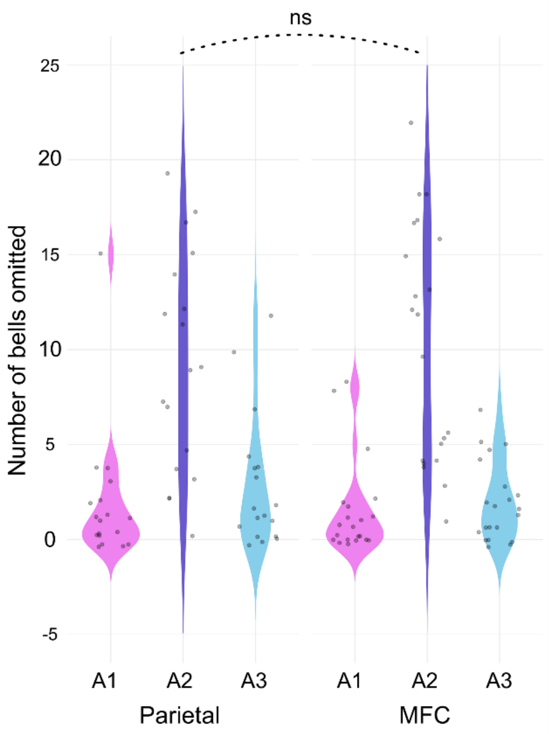

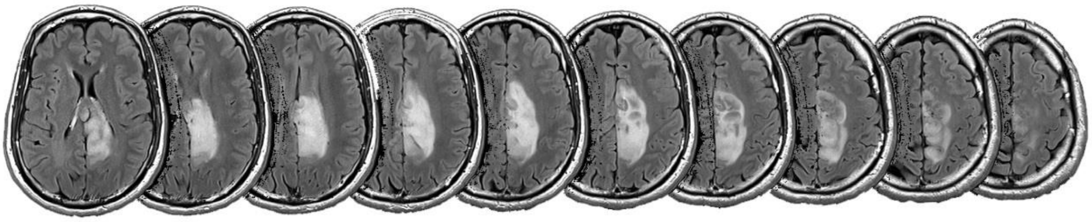

**Supplementary Figure 6:** Native FLAIR MRI of the unique patient. As shown in this figure. The tumor (lower-grade glioma) mainly infiltrated the right cingulum.

## 2- Supplementary Tables

**Supplementary Table 1:** Sociodemographic and clinical data for the 128 patients

| Variables | mean | SD | range |
|---|---|---|---|
| Age (years) | 39.71 | ± 12.34 | [20; 70] |
| Educational attainment (full-time years) | 14.75 | ± 2.85 | [2; 22] |
| Gender (n) | F: 54 | M: 74 | |
| Handedness (n) | L: 3 | A: 4 | R: 121 |
| Preoperative volume ($cm^3$) | 57.49 | ± 48.98 | [4.27; 239.69] |
| Postoperative volume ($cm^3$) | 47.20 | ± 39.72 | [2.24; 195.09] |

F, female; M, male; L, left-handed; R, right-handed; A, ambidextrous. In brackets, range.

**Supplementary Table 2**: Correlation analyses between the behavioral measurements of visuo-spatial attention and the demographic/clinical data

| | | **age** | **educational attainment** | **lesion volume*** |
|---|---|---|---|---|
| **line bisection** | A1 | -0.02 | -0.12 | 0.01 |
| | Δ*1* | -0.08 | 0.00 | -0.11 |
| | Δ*2* | -0.09 | 0.00 | 0.13 |
| **total_bell** | A1 | -0.06 | -0.06 | 0.14 |
| | Δ*1* | 0.13 | 0.07 | -0.02 |
| | Δ*2* | **0.25** | 0.002 | 0.16 |
| **diff_bell** | A1 | 0.00 | 0.05 | -0.10 |
| | Δ*1* | -0.05 | 0.05 | -0.13 |
| | Δ*2* | -0.16 | 0.16 | 0.05 |

Spearman correlations were performed to assess the relationships between the behavioral measurements and the demographic and clinical data. The results are not adjusted for multiple comparisons. Only one slight but significant correlation (bold) was found between *age* and *total_bell (*Δ*2)*. * Lesion volume corresponds to preoperative tumor volumes for A1 and to postoperative resection cavity volumes for A2, A3, delta1 and delta2.

**Supplementary Table 3**: Longitudinal behavioral data for the 128 patients

| | **A1** *(n = 128)* | | **A2** *(n = 128)* | | **A3** *(n = 128)* | |
|---|---|---|---|---|---|---|
| | mean ± SD | range | mean ± SD | range | mean ± SD | range |
| **Line Bisection** (deviation in mm) | -0.98 ± 2.21 | [-6.8; 6.0] | 0.94 ± 5.70 | [-8.1; 34.7] | -0.81 ± 3.42 | [-9.0; 17.1] |
| ***Total_bell*** (omitted bells) | 2.19 ± 2.61 | [0; 15] | 5.59 ± 5.24 | [0; 22] | 2.04 ± 2.33 | [0; 12] |
| ***Left_bell*** (omitted bells) | 1.15 ± 1.59 | [0; 8] | 3.96 ± 4.44 | [0; 17] | 1.15 ± 1.60 | [0; 8] |
| ***Right_bell*** (omitted bells) | 1.11 ± 1.44 | [0; 10] | 1.60 ± 1.61 | [0; 7] | 0.92 ± 1.15 | [0; 5] |
| ***Diff_bell*** (left minus right) | 0.04 ± 1.48 | [-5; 4] | 2.35 ± 4.17 | [-4; 18] | 0.22 ± 1.52 | [-3; 6] |

**Supplementary Table 3** (continued)

| **Delta_1** (A2 minus A1) | | **Delta_2** (A3 minus A1) | |
|---|---|---|---|
| mean ± SD | range | mean ± SD | range |
| 1.92 ± 5.38 | [-8.0; 35.5] | 0.18 ± 3.01 | [-13.8; 17.9] |
| 3.39 ± 5.84 | [-13; 22] | -0.16 ± 2.94 | [-12; 12] |
| 2.80 ± 4.77 | [-4; 18] | 0 ± 1.90 | [-6; 8] |
| 0.49 ± 2.15 | [-10; 5] | -0.18 ± 1.67 | [-10; 4] |
| 2.31 ± 4.36 | [-4; 20] | -0.17 ± 2.07 | [-5; 8] |

Source data are provided as a Source Data file.

**Supplementary Table 4**: demographic and behavioral data for the 44 healthy participants

| | **mean** | **SD** | **range** |
|---|---|---|---|
| **Age** | 38.34 | 11.26 | [19; 68] |
| **Educational attainment (full-years)** | 15.27 | 2.67 | [19; 20] |
| **Sex** | 20 Females; 24 Males | | |
| **handedness** | all right-handed | | |
| **Line bisection** | -2.41 | 2.77 | [-7.3; 4.3] |
| **Total_bell** | 1.22 | 1.51 | [0; 5] |
| **Diff_bell** | 0.16 | 1.27 | [-3; 4] |

Source data are provided as a Source Data file.

**Supplementary Table 5**: Comparisons between controls and patients at each assessment

| Time point | Measurements | $t_{(170)}$ | *p* | diff in mean | - CI(95) | +CI(95) |
|---|---|---|---|---|---|---|
| **A1** | bisection line | -3.45 | **< 0.001** | -1.43 | -2.25 | -0.61 |
| | total_bell | -2.32 | **0.021** | -0.97 | -1.79 | -0.15 |
| | diff_bell | 0.48 | 0.63 | 0.12 | -0.37 | 0.61 |
| **A2** | bisection line | -3.74 | **< 0.001** | -3.35 | -5.12 | -1.58 |
| | total_bell | -5.43 | **< 0.001** | -4.37 | -5.95 | -2.78 |
| | diff_bell | -3.43 | **< 0.001** | -2.19 | -3.46 | -0.93 |
| **A3** | bisection line | -2.81 | **0.006** | -1.61 | -2.73 | -0.48 |
| | total_bell | -2.15 | **0.033** | -0.81 | -1.56 | -0.07 |
| | diff_bell | -0.23 | 0.82 | -0.06 | -0.56 | 0.44 |

Two-sided *t*-tests were used to determine statistical difference between the control and the patient group for each behavioral measurement and for each assessment. Note that two-sided non-parametric statistics (Mann-Whitney) lead to the same results (See Supplementary Analyses). Bold means statistical significance. Source data are provided as a Source Data file.

**Supplementary Table 6**: Between-task correlations

***A1***

| | line bisection | total_bell | diff_bell |
|---|---|---|---|
| **line bisection** | 1 | | |
| **total_bell** | 0.01 (ns) | 1 | |
| **diff_bell** | **0.23** (*p* = 0.009) | 0.07 (ns) | 1 |

***Δ1***

| | line bisection | total_bell | diff_bell |
|---|---|---|---|
| **line bisection** | 1 | | |
| **total_bell** | **0.37** (*p* < 0.001) | 1 | |
| **diff_bell** | **0.25** (*p* < 0.001) | **0.44** (*p* < 0.001) | 1 |

***Δ2***

| | line bisection | total_bell | diff_bell |
|---|---|---|---|
| **line bisection** | 1 | | |
| **total_bell** | 0.13 (ns) | 1 | |
| **diff_bell** | 0.14 (ns) | 0.06 (ns) | 1 |

Spearman's rank coefficient correlation (two-sided) is indicated in each case. Bold represents significance at $p < 0.05$ uncorrected. Only slight but significant correlations were found between the two lateralized measures of visuo-spatial attention (i.e. *line bisection* and *diff_bell*). ns, non-significant. Source data are provided as a Source Data file.

**Supplementary Table 7**: Proportion of parietal and MFC patients with a new deficit after surgery

| | **Parietal Group** | | | | **MFC Group** | | | |
|---|---|---|---|---|---|---|---|---|
| | deficit A1 | | new deficit A2 | | deficit A1 | | new deficit A2 | |
| | *n* | % | *n* | % | *n* | % | n | % |
| Bisection line | 1 | 5.5 | 11 | 61.1 | 1 | 4.7 | 3 | 14.3 |
| total_bell | 3 | 16.7 | 13 | 72.2 | 3 | 14.3 | 16 | 76.2 |
| diff_bell | 1 | 5.5 | 8 | 44.4 | 1 | 4.7 | 14 | 66.7 |

In this table, it is shown the proportion of patients with a pathological deviation at *A1* (z-score ≥ 1.65) and the proportion of patients with a new deficit at *A2* (i.e. patients for whom the performance was unimpaired prior to surgery). The proportion of new deficits is relatively comparable between both groups for *total_bell* (2-tailed $\chi^2 = 0.08$, $p = 0.78$) and *diff_bell* (2-tailed $\chi^2 = 1.95$, $p = 0.78$), but unequal for *line bisection* estimates (2-tailed $\chi^2 = 9.23$, $p = 0.0024$). This is in agreement with the results from the between-group analyses described in the main text.

## 3- Supplementary analyses

In this study, parametric statistics were used to assess between-group and within-subject differences despite the commonly observed nonnormality of neuropsychological data. This approach was preferred given the large sample size and to allow the assessment of interaction effects. Here we provide the corresponding two-sided nonparametric analyses, except for the mixed ANOVA used to test the interaction effect between time of assessment and patient groups (section 'group analyses'). Note that the results are strictly the same.

(1) Comparisons between the control group and the patient group at each assessment (A1, A2 and A3).

| **Mann-Whitney U-test** | **U** | **Z** | ***p*** |
|---|---|---|---|
| *Controls versus patients (A1)* | | | |
| Line bisection | 1935 | -3.09 | 0.002 |
| total_bell | 2207.5 | -2.14 | 0.028 |
| diff_bell | 2599.5 | 0.76 | 0.42 |
| *Controls versus patients (A2)* | | | |
| Line bisection | 1630 | -4.16 | < 0.001 |
| total_bell | 1092 | -6.05 | < 0.001 |
| diff_bell | 1807 | -3.54 | <0.001 |
| *Controls versus patients (A3)* | | | |
| Line bisection | 2019.5 | -2.79 | 0.005 |
| total_bell | 2243.5 | -2.01 | 0.045 |
| diff_bell | 2793.5 | 0.08 | 0.94 |

(2) Behavioral differences between each assessment (A1, A2 and A3)

- Non parametric ANOVAs

| | Friedman ANOVA | | | |
|---|---|---|---|---|
| | *n* | df | $X^2$ | *p* |
| Line bisection | 128 | 2 | 10.27 | 0.006 |
| total_bell | 128 | 2 | 60.41 | <0.001 |
| diff_bell | 128 | 2 | 33.67 | <0.001 |

df = degree of freedom.

- Post-hoc analyses

| | Tukey's test for pairwise multiple comparisons | | | | | | | | |
|---|---|---|---|---|---|---|---|---|---|
| | A1 vs A2 | | | A2 vs A3 | | | A1 vs A3 | | |
| | Diff of ranks | q | p | Diff of ranks | q | p | Diff of ranks | q | p |
| Line bisection | 42.5 | 3.76 | <0.05 | 44.5 | 3.93 | <0.05 | 2 | 0.18 | ns |
| total_bell | 85 | 7.56 | <0.05 | 108 | 9.55 | <0.05 | 22.5 | 1.99 | ns |
| diff_bell | 78 | 6.89 | <0.05 | 63 | 5.57 | <0.05 | 15 | 1.32 | ns |

(3) Behavioral differences between ∆1 and ∆2

| | Wilcoxon test | | | |
|---|---|---|---|---|
| | n | T | Z | P |
| Line bisection | 128 | 2435 | 3.7 | <0.001 |
| total_bell | 128 | 476 | 7.37 | <0.001 |
| diff_bell | 128 | 961.5 | 5.28 | <0.001 |

## 4- Supplementary Notes

**Supplementary Note 1.** In the discussion section of the article, we mentioned that the fibers of SLF_1 were *likely* to be damaged in the single patient after the first surgery, while the disconnection analysis indicated that the fibers were almost completely interrupted. We interpreted this result with caution in view of the method used to estimate disconnection severity. The measures of disconnection computed by the LQT toolbox is based on fiber tracts generated from the averaged diffusion data of the 1065 HCP participants. As comprehensively discussed by Griffis et al.[1], the clear advantage is to deal with tracts that represent the most commonly shared features of white matter architecture at the population level and that are constructed on the basis of an unprecedented sample of individual data. The counterpart is that the interindividual variability in the spatial arrangement of tracts are not directly taken into consideration in the disconnection analyses. As a consequence, if the SLF_I of the single patient has an “outlier” spatial distribution, we cannot formally exclude the

possibility that the tract is less affected in reality. While the disconnection results of the single patient does not allow to provide strong conclusion about the role of this tract in line bisection deficits, this does not alter the suitability of the interpretation we proposed. If damage to SLF_I is a central mechanism underlying the occurrence of line bisection deficits, then such deficits are expected to vastly occur in the MPF group because the cortical projections of SLF_I mainly target SMA, pre-SMA, anterior/middle cingulate and medial SFG[2–4]. Our results did not reveal such a behavioral pattern.

**Supplementary Note 2.** It is interesting to note that neglect symptoms were long-lasting in the single patient following the second surgery as opposed to transient (or significantly recovered) in most patients having undergone a single stage surgery. While the current data does not allow to provide a clear-cut interpretation for this lack of recovery which is likely to be multidetermined, several lines of explanations can be offered. First, no mapping of visuo-spatial attention was performed with the line bisection task and we used a trans-cortical surgical approach through the anterior precuneus/SPL to access the posterior part of the tumor. Accordingly, the identified perceptive neglect might arise from a topological mechanism involving this cortical region. Second, beyond the SLF_I/ cingulum disconnection which was likely to already occur following the first surgery, damage to other tracts was observed, mainly including EMC and the middle-to-posterior part of the corpus callosum, and less severely the middle longitudinal fasciculus, parieto-pontine tract, medial lemniscus, posterior thalamic radiations and posterior cortico-striatal tract (see main text). As a result, a complex pattern of disconnection might also account for the lasting neglect. Last, the cumulative damage to the medial network (both cortically and subcortically) following both surgeries might severely diminish the possibility to initiate efficient strategies of functional reorganization, resulting in permanent neglect signs.

**Supplementary Note 3.** Disconnection analyses were performed in this study to ascertain the extent to which surgical resections could account for the occurrence of neglect signs in the short and longer term. A note of caution should be clearly mentioned here. The patient sample on which we relied was uniquely composed of patients harboring a lower-grade glioma. Although compared to higher grade glioma mass effect is much less frequent in this tumor grade, it may nevertheless occur in certain patients and consequently bias the expected spatial positioning of white matter tracts (and thus the measures of disconnection severity). However, we are relatively protected from this potential shortcoming as disconnection analyses were uniquely performed on the basis of resection cavity maps (if any, the mass effect is released at this stage). Moreover, the sample size – which is relatively large - allows to smooth, at least to some extent, the effect of this potential bias on the final results.

## 5- Supplementary References


1. Griffis. J. C., Metcalf. N. V., Corbetta. M. & Shulman. G. L. Lesion Quantification Toolkit: A MATLAB software tool for estimating grey matter damage and white matter disconnections in patients with focal brain lesions. *BioRxiv* (2020).
2. Makris, N. *et al.* Segmentation of subcomponents within the superior longitudinal fascicle in humans: a quantitative. in vivo. DT-MRI study. *Cerebral Cortex* **15**. 854–869 (2005).

3. Rojkova, K. *et al.* Atlasing the frontal lobe connections and their variability due to age and education: a spherical deconvolution tractography study. *Brain Structure and Function* **221**. 1751–1766 (2016).
4. Komaitis, S. *et al.* Dorsal component of the superior longitudinal fasciculus revisited: novel insights from a focused fiber dissection study. *Journal of Neurosurgery* **132**. 1265–1278 (2019).
