# Supplementary material for: Contribution of the medial eye field network to the voluntary deployment of visuospatial attention
Source: Nat Commun. 2022 Jan 17;13:328. doi: 10.1038/s41467-022-28030-3 (PMC8763913; doi:10.1038/s41467-022-28030-3)
Supplement: Supplementary file 3 — Reporting summary [file 41467_2022_28030_MOESM3_ESM.pdf]

nature portfolio

Corresponding author(s): Guillaume HERBET

Last updated by author(s): 05/12/2021

Reporting Summary

Nature Portfolio wishes to improve the reproducibility of the work that we publish. This form provides structure for consistency and transparency in reporting. For further information on Nature Portfolio policies, see our Editorial Policies and the Editorial Policy Checklist.

Statistics

For all statistical analyses, confirm that the following items are present in the figure legend, table legend, main text, or Methods section.

n/a	Confirmed	
☐	☒	The exact sample size (n) for each experimental group/condition, given as a discrete number and unit of measurement
☐	☒	A statement on whether measurements were taken from distinct samples or whether the same sample was measured repeatedly
☐	☒	The statistical test(s) used AND whether they are one- or two-sided<br>*Only common tests should be described solely by name; describe more complex techniques in the Methods section.*
☒	☐	A description of all covariates tested
☐	☒	A description of any assumptions or corrections, such as tests of normality and adjustment for multiple comparisons
☐	☒	A full description of the statistical parameters including central tendency (e.g. means) or other basic estimates (e.g. regression coefficient) AND variation (e.g. standard deviation) or associated estimates of uncertainty (e.g. confidence intervals)
☐	☒	For null hypothesis testing, the test statistic (e.g. F, t, r) with confidence intervals, effect sizes, degrees of freedom and P value noted<br>*Give P values as exact values whenever suitable.*
☒	☐	For Bayesian analysis, information on the choice of priors and Markov chain Monte Carlo settings
☒	☐	For hierarchical and complex designs, identification of the appropriate level for tests and full reporting of outcomes
☐	☒	Estimates of effect sizes (e.g. Cohen's d, Pearson's r), indicating how they were calculated

Our web collection on statistics for biologists contains articles on many of the points above.

Software and code

Policy information about availability of computer code

Data collection	No specific codes were used to acquire the data analysed in this study.
Data analysis	SVR-LSM analyses were performed with the Matlab code by Zhang et al. (https://github.com/yongsheng-zhang/SVR-LSM). Optimization of SVR-LSM hyper-parameters was performed with the Matlab code by Wiesen et al. (https://data.mendeley.com/datasets/2hyhk44zrj/2). Measures of disconnection severity were performed with Lesion Quantification Toolkit (Griffis et al., 2021) (https://wustl.box.com/v/LesionQuantificationToolkit). The three softwares were run under Matlab (version 2020b). Violin and notch boxplots were made with R-software (Version 1.4.1103)

For manuscripts utilizing custom algorithms or software that are central to the research but not yet described in published literature, software must be made available to editors and reviewers. We strongly encourage code deposition in a community repository (e.g. GitHub). See the Nature Portfolio guidelines for submitting code & software for further information.

Data

Policy information about availability of data

All manuscripts must include a data availability statement. This statement should provide the following information, where applicable:

- Accession codes, unique identifiers, or web links for publicly available datasets
- A description of any restrictions on data availability
- For clinical datasets or third party data, please ensure that the statement adheres to our policy

The unthresholded SVR-LSM p-maps, the raw beta maps, the raw lesion maps and the overlap maps generated in this study as well as the raw behavioral dataset are available without restriction at https://doi.org/10.6084/m9.figshare.16822306.v3. All raw behavior data are also provided in the Source data files. The 1065-HCP tractography atlas is available at https://brain.labsolver.org/.

Field-specific reporting

Please select the one below that is the best fit for your research. If you are not sure, read the appropriate sections before making your selection.

☒ Life sciences ☐ Behavioural & social sciences ☐ Ecological, evolutionary & environmental sciences

For a reference copy of the document with all sections, see nature.com/documents/nr-reporting-summary-flat.pdf

Life sciences study design

All studies must disclose on these points even when the disclosure is negative.

Sample size	The sample size was n = 128 patients. No sample size calculation was performed: we relied on the largest longitudinal dataset ever published to our knowledge gained from homonegous patients with a lower-grade glioma - a rare tumor of the CNS. Previous studies have shown that a sample size of 100-120 patients is enough to provide stable lesion-symptom results in the context of SVR-LSM (e.g. Wiesen et al., 2019; Ivanova et al., 2021). As healthy controls were retrospectively enrolled, the quota method (based on age, educational attainment and sex) was used to match the control group with the patient group. No sample size calcuation was performed; we considered that n = 44 control participants was enough to adequately represent the inter-individual variability.
Data exclusions	No data were exluded
Replication	A 5-fold cross-validation procedure was used to estimate prediction accuracy and reproducibility of SVR-LSM models
Randomization	Not relevant here: just one experimental group included in the main analyses.
Blinding	no group allocation.

Reporting for specific materials, systems and methods

We require information from authors about some types of materials, experimental systems and methods used in many studies. Here, indicate whether each material, system or method listed is relevant to your study. If you are not sure if a list item applies to your research, read the appropriate section before selecting a response.

Materials & experimental systems

n/a	Involved in the study	
☒	☐	Antibodies
☒	☐	Eukaryotic cell lines
☒	☐	Palaeontology and archaeology
☒	☐	Animals and other organisms
☐	☒	Human research participants
☒	☐	Clinical data
☒	☐	Dual use research of concern

Methods

n/a	Involved in the study	
☒	☐	ChIP-seq
☒	☐	Flow cytometry
☐	☒	MRI-based neuroimaging

Human research participants

Policy information about studies involving human research participants

Population characteristics	The patient sample consisted of 128 patients (mean age: 39.7 ± 12.3, 54 females; 121 right-handed) consecutively operated on for a lower grade glioma. The control group was composed of 44 neurologically healthy participants (mean age: 38.3 ± 11.3, 20 females; all right-handed).
Recruitment	All patients operated on in our institution for a lower-grade glioma infiltrating the right hemisphere between 2013 and 2020 was included. Patients fulfilling the following exclusion criteria were discarded at the outset: higher-grade glioma identified by histopathological analyses, adjuvant radiotherapy performed before or after surgery, a visual hemianopsia identified before or after surgery to avoid contaminating task performance, and a lack of longitudinal behavioral data. As healthy controls were retrospectively enrolled, the quota method (based on age, educational attainment and sex) was used to match the control group with the patient group.
Ethics oversight	All participants provided informed consent.<br>Montpellier University Medical center's institutional review board for patients<br>French College of Neurosurgery for healthy participants

Note that full information on the approval of the study protocol must also be provided in the manuscript.

Magnetic resonance imaging

Experimental design

Design type	Support vector regresion-lesion-symptom mapping (SVR-LSM) and disconnection analyses
Design specifications	Not relevant here: no in-scanner tasks were performed by the participants.
Behavioral performance measures	Two tasks tapping into the process of visuo-spatial attention (i.e. the line bisection task and the bell test) at three time points (i.e. before surgery, 4 days after and three months after surgery). We used as dependent variables the preoperative performances, the behavioral difference between the preoperative and the 4-day postoperative assessment and the behavioral difference between the preoperative and the 3-month postoperative assessment.

Acquisition

Imaging type(s)	3D lesion maps drawn from normalized structural MRIs
Field strength	1.5T or 3T
Sequence & imaging parameters	The imaging parameters were as follows: (i) preoperative FLAIR images (1.5T/3T): repetition time, 13200/800 ms; echo time,109/108 ms; inversion time, 2500/23700 ms; field of view 210 × 240/202 × 240 mm, voxel size 0.898 × 0.898 × 6 mm3, slice thickness 5/3 mm, spacing 5.5/3.6 mm, and flip angle 150°; (ii) 3-month 3DT1 images (1.5T/3T): repetition time, 1880/1700 ms; echo time, 3.4/2.5 ms; inversion time, 1100/922 ms; field of view, 256 × 256 mm; voxel size, 1 × 1 × 1 mm3, 176 axial slices, and flip angle 15°/9°.
Area of acquisition	Brain areas lesionned in at least three patients

Diffusion MRI ☐ Used ☒ Not used

Preprocessing

Preprocessing software	SPM12 (last version), Clinical Toolbox (last version)
Normalization	Enantiomorphic normalization using SPM12' Clinical Toolbox (last version)
Normalization template	MNI152
Noise and artifact removal	To minimize the potential bias caused by abnormal lesion-related radiological signals, MRI datasets were registered to the MNI space using enantiomorphic normalization
Volume censoring	Not relevant here: we did not process fMRI data.

Statistical modeling & inference

Model type and settings	Multivariate for lesion-symptom analyses and univariate for disconnection analyses
Effect(s) tested	We assessed the relationship between lesion locations and behavior outcomes (SVR-LSM) and the relationship between disconnection severity and behavioral outcomes (disconection analyses)

Specify type of analysis: ☒ Whole brain ☐ ROI-based ☐ Both

Statistic type for inference (See Eklund et al. 2016)	Permutation procedure (5000) to derive p-maps from raw beta maps
Correction	FDR at q = 0.05

Models & analysis

n/a	Involved in the study
☒	☐ Functional and/or effective connectivity
☒	☐ Graph analysis
☐	☒ Multivariate modeling or predictive analysis

Multivariate modeling and predictive analysis	We used support vector regression to model the relationships between damaged voxels and behavioral data outcomes.
